# Gene Expression Changes Between Patent and Fused Cranial Sutures in a Nonsyndromic Craniosynostosis Population

**Published:** 2015-04-10

**Authors:** Amiee B. Potter, Jennifer L. Rhodes, Rafael A. Vega, Thomas Ridder, Rita Shiang

**Affiliations:** ^a^Integrated Genomics Laboratory, Oregon Health & Science University, Portland; ^b^Division of Plastic & Reconstructive Surgery, Department of Surgery, Virginia Commonwealth University Health System, Richmond; ^c^Department of Neurosurgery, Virginia Commonwealth University Health System, Medical College of Virginia, Richmond; ^d^Department of Human and Molecular Genetics, Virginia Commonwealth University, Richmond

**Keywords:** craniosynostosis, osteoblastogenesis, calvaria, sutures, gene expression

## Abstract

**Objective:** Craniosynostosis is a premature fusion of 1 or more cranial sutures. It may occur with additional morphological abnormalities (syndromic) or in isolation. Studies suggest that dysregulation of normal cell proliferation, differentiation, and migration has a role in isolated or nonsyndromic craniosynostosis but the molecular mechanisms remain unknown. The aim of this research is to identify genes differentially expressed in prematurely fused human suture compared to patent suture in nonsyndromic craniosynostosis. **Methods:** Bone fragments from synostosed and patent sutures of 7 infants with nonsyndromic craniosynostosis were collected during surgical release of fused sutures. RNA was isolated from the fragments (7 patent and 7 fused) and global gene expression profiled using the Illumina WGE-DASL assay and HumanRef 8.0 Beadchip. **Results:** Comparison of mRNA expression in fused and patent suture identified 68 genes significantly differentially expressed and having fold changes ≤ −2.0 and ≥ 2.0 with a false discovery rate adjusted *P* value at .10 and 136 with adjusted *P* value of 0.15. *SFRP2* (secreted frizzled-related protein 2) demonstrated the largest decrease in fused sutures. Analysis including only sagittal fused sutures revealed a set of 35 overlapping genes that may be involved in suture patency over all suture types. *SPHKAP* (sphingosine kinase type 1-interacting protein), a modulator of *TGFβ* signaling, was significant in the sagittal subset. **Conclusion:** Differentially expressed genes were identified in fused suture relative to patent in a nonsyndromic craniosynostosis population. *SFRP2* is likely important in suture patency. Genes having significant roles in osteoblastogenesis as negative regulators of canonical Wnt pathway were significantly downregulated.

Craniosynostosis, premature fusion of 1 or more cranial sutures, occurs 1 in 2000 live births. The developing cranium is prevented from expanding in a direction perpendicular to the fused suture. This can potentially lead to orbitofacial anomalies as well as cognitive, speech, visual, and social impairment. Eighty-five percent of cases of craniosynostosis occur as isolated pathology, termed nonsyndromic craniosynostosis (NSC), while the remaining cases are seen along with a suite of morphological abnormalities, craniofacial and postcranial, caused by mutations within specific genes and characteristic of syndromes.

Cranial suture biology includes complex, intricate, and highly coordinated processes that are mediated by tightly regulated gene expression.^1^^-^5 Mutations have been identified within a number of genes associated with both syndromic and nonsyndromic forms of craniosynostosis.^6^ More recently, microarray analysis has been used to examine gene expression in both open and prematurely closed sutures, as well as in in vitro systems using cell culture.^7^^-^9 Holmes and Basilico^10^ demonstrated that in a mouse model expressing the S252W mutation in *FGFR2* associated with Apert syndrome. Previous studies have combined samples from individuals with syndromic and nonsyndromic forms of craniosynostosis to identify differentially expressed genes. Animal studies used models with known human mutations in the syndromic form of the disorders for their studies. While gene mutations may ultimately prove causative, studies suggest that the proximate mechanisms leading to abnormal suture closure may be downstream events affecting cell signaling.^1^^,^^7^^,^^8^^,^^11^

There is growing evidence that suggests that premature suture closure is the result of acceleration and disruption of the normal timing and balance of cell proliferation, differentiation, and migration, respectively.^1^^,^^3^^,^^6^^,^^11^ Furthermore, the osteoblasts within the osteogenic front may play a significant role in premature fusion rather than the underlying dura.^10^ Therefore, we hypothesize that the pattern of gene expression within the osteogenic front of the closed/closing suture will differ from the open suture with dysregulation of genes regulating osteoblast progenitor cell differentiation and proliferation, and osteoblast maturation playing a significant role in the pathogenesis of isolated craniosynostosis. The aim of this research was to identify which genes are differentially expressed in prematurely fused human suture compared to patent suture in only NSC in an effort to discern which genes are dysregulated at the osteogenic front and to determine whether downstream changes are similar to other studies. We used only primary suture tissue from a group of 7 individuals with only NSC of mainly sagittal but also including 1 metopic and coronal fused suture and no known gene mutations to study differential gene expression.

## METHODS

### Samples

Prior to study enrollment, all patients were examined by a medical geneticist for determination of the status of their craniosynostosis as syndromic or nonsyndromic. Children with only a single suture that was fused, without malformations in other organ systems and molecular genetic testing for *FGFR 1, 2, 3* and *TWIST* mutations failed to reveal any mutation, were considered nonsyndromic. Written informed consent was obtained from the parents of 7 infants diagnosed with NSC prior to initial craniosynostosis surgery. The study protocol was approved by the institutional review board at Virginia Commonwealth University. All sutures were completely fused, which was verified with computed tomographic scans preoperatively and by observation intraoperatively. Bone fragments were recovered from synostosed and patent cranial sutures of each patient undergoing surgical correction. Using a 2-mm Kerrison ronqeur, samples were taken within 1 mm of the location where the ectocranial ridging of the fused suture was most prominent. Patent samples were taken with the same instrument across the patent suture to include no more than 1 mm from the open suture (Supplemental Figure 1S). The fragments were immediately stored in RNA later (Qiagen) solution and then kept at −80°C until ready for isolation. The synostosed fragments of each participant were kept separate from the patent sutural material. Therefore, 14 total samples were included in the study: 7 synostosed and 7 patent samples. The synostosed sutures were derived from 5 sagittal, 1 unilateral coronal and 1 metopic. Participants included male and female infants younger than 1 year (Table 1).

### RNA isolation, quality, and integrity

Frozen bone was pulverized and homogenized in TRIzol reagent (Life Technologies). A standard TRIzol protocol was followed to isolate RNA followed by isopropanol precipitation and 70% ethanol wash (http://tools.invitrogen.com/content/sfs/manuals/trizol_reagent.pdf). Purity and concentration of the total RNA were tested using a Nanodrop spectrophotometer. The A260/A280 ratio ranged from 1.45 to 1.95 (average = 1.7). Integrity of the RNA was determined using the Agilent 2100 Bioanalyzer that indicated RNA degradation with RINs ranging from 2.0 to 6.80 (average 4.0). Thus, the Illumina WGE-DASL assay was chosen for this study as it is designed specifically for degraded RNA expression profiling. RNA was not pooled for this study.

### Gene expression, preliminary data analysis, and statistical methods

Global gene expression was measured using the Illumina WGE-DASL BeadChip Assay. Reactions were performed according to the manufacturer's WGE-DASL Manual (Illumina). In brief, total RNA was used to generate cDNA using biotinylated oligo-dT18 and random primers. Pooled assay specific oligonucleotides targeting a 50 nucleotide sequence of each cDNA and provided in the WGE-DASL assay pool were annealed to the targeted cDNAs during a 16-hour incubation. This was followed by enzymatic extension and ligation steps, as described in the manufacturer's protocol. Ligated products were PCR amplified and labeled with a universal Cy3-coupled primer after which single-stranded labeled products were precipitated and then hybridized to HumanRef-8 expression BeadChips (Illumina). The bead arrays were washed and coated. Finally, the raw expression intensities, as measured by fluorescence, were captured, by scanning the arrays, on a BeadArray Reader (Illumina).

Raw image files were imported into GenomeStudio (Illumina). All BeadArray data were normalized using the Quantile method without background subtraction. Overall hybridization and array performance were assessed using GenomeStudio QA tools. Normalized annotated sample results were exported in .txt format and imported into Partek Genomics Suite (Build 6.6) for identification of differentially expressed mRNA transcripts.

Changes in mRNA transcript expression were assessed for the synostosed sutures using intensity data of RNA derived from patent suture samples as baseline. Therefore, we compared the gene expression in synostosed sutures to patent suture. Genes were considered to have increased or decreased expression in the prematurely fused suture set relative to the patent suture set. The paired *t* test was used to determine differentially expressed genes with significance set at ≤.05 and the Benjamini and Hochberg correction for multiple testing was conducted with adjusted *P* value set at .10. To refine the gene list, fold changes were calculated by using the unlogged intensities to calculate the ratio of change in synostosed suture when using patent suture expression as baseline or control. Genes significant with false discovery rate (FDR) of 0.10 or smaller and fold changes FDR and fold changes (ratios) −2.0 or less and 2.0 or more were retained for further investigation and are referred to as our set of FDR 0.10 genes. Genes were observed approaching significance with FDR of 0.10 and fold changes (ratios) −2.0 or less and 2.0 or more. Therefore, the FDR was relaxed to 0.15 to include these genes. This set, which includes the FDR 0.10 genes, is referred to as our FDR 0.15 genes. We also conducted paired *t* test on a subset of the samples contributed only from patients with sagittal synostosis. Both males and females were included in the study. Synostosed suture derived from the prematurely fused sagittal suture while the patient's paired patent suture control derived from lambdoidal suture. Total sample size was 10: 5 synostosed and 5 patent. Again, baseline expression was determined in patent sutures with change in expression in synostosed suture relative to this. Analysis was performed using Partek software and the paired *t* test subroutine. Significance was set at *P* ≤ .05 with FDR at 0.10 to correct for multiple testing. We also considered a qualitative method of calculating fold changes. Genes significant with FDR 0.10 were then further filtered by implementing a fold change stringency of −2.0 or less and 2.0 or more to the list. We also applied the fold change stringency to the list of significantly differentially expressed genes with relaxed FDR of 0.15. All microarray data have been submitted to the Gene Expression Omnibus database at NCBI with the accession number GSE50796.

### Validation

To confirm global gene expression analysis using the Illumina WGE-DASL BeadChip platform, qRT-PCR assay was performed for 4 genes from our gene lists. Reverse transcription of total RNA using the SYBR Green qPCR reagents (Applied Biosystems Inc) was performed. Specifically, first strand cDNA was generated from 2.5 μg of total RNA with 5 ng/μL of oligo dT and random primers and 0.5 mM dNTPs. The reaction was heated to 65°C for 5 minutes and placed on ice. First strand reaction buffer (1X), 0.01 M DTT, and 40 U of RNase inhibitor (Promega) were added and the reaction was incubated at 37°C for 2 minutes. MMLV-RT was added (200 U) (Invitrogen) and the sample was incubated at 37°C for 1 hour followed by a final incubation of 95°C for 5 minutes. qRT-PCR for validation of gene expression changes was performed in triplicate using the Applied Biosystems (ABI) 7500 Fast Real Time System. Primers were designed and optimized using standard PCR. Reactions were set up using 4 μL of cDNA diluted 1:8, 3 pmol/μl primers and 10 μL of 2X SYBR Green qPCR Master Mix (ABI) in a total volume of 20 μL. A 2-step PCR protocol was used with a 10-minute denaturation step at 95°C and then 40 cycles of 95°C for 15 seconds and 60°C for 1 minute. A melt curve analysis was performed to ensure PCR specificity. For each experimental sample, 18S was also run as an internal control. Ribosomal 18S RNA is a common housekeeping gene widely used as an endogenous control including in other studies using human sutures.^7^ Output was analyzed using SDS 7500 Fast software version v1.3.1. Values were obtained using a standard curve of dilutions of cDNA derived from RNA isolated from normal bone and normalized with *18S*. The relative standard curve method was used to analyze the expression results and used to calculate fold changes. Table 2 presents the genes tested, primer sequences, and expected amplicon length for the validation experiments (Supplementary Figure 2S).

### Functional analysis

**Figure 1:**
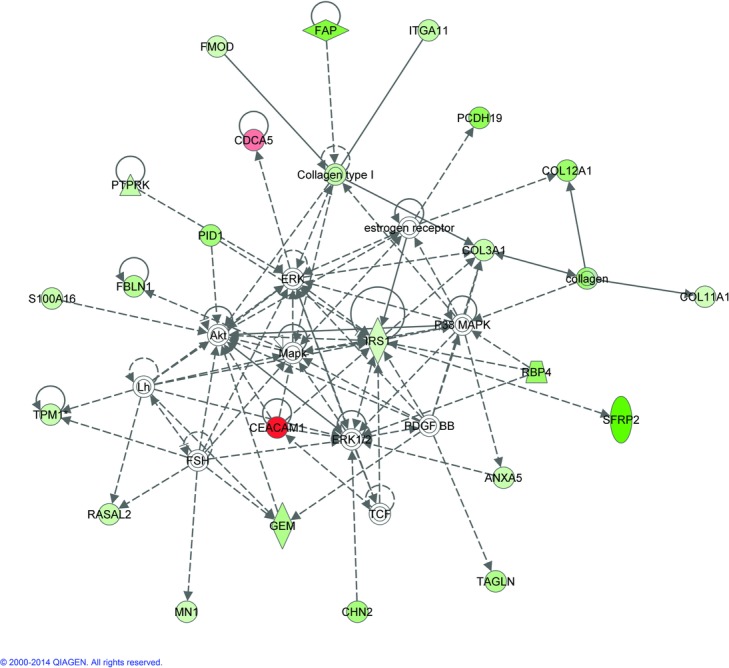
Pathway analysis. De novo pathway analysis using the false discovery rate (FDR) 0.10 gene list. Pathways were built with Ingenuity Pathway Analysis taking the genes with significant gene expressions changes from this experiment and identifying known relationships between the genes. Green indicates genes downregulated and red indicates upregulated genes from this study. The white genes did not have significant gene expression changes but link the genes in the network that are significantly changed. The intensity of the color indicates the degree of the expression change. Solid lines indicate direct interaction, while dashed lines indicate indirect interaction. Lines indicate binding between molecules, while arrows indicate molecule that can act on another which may or may not also bind.

**Figure 2:**
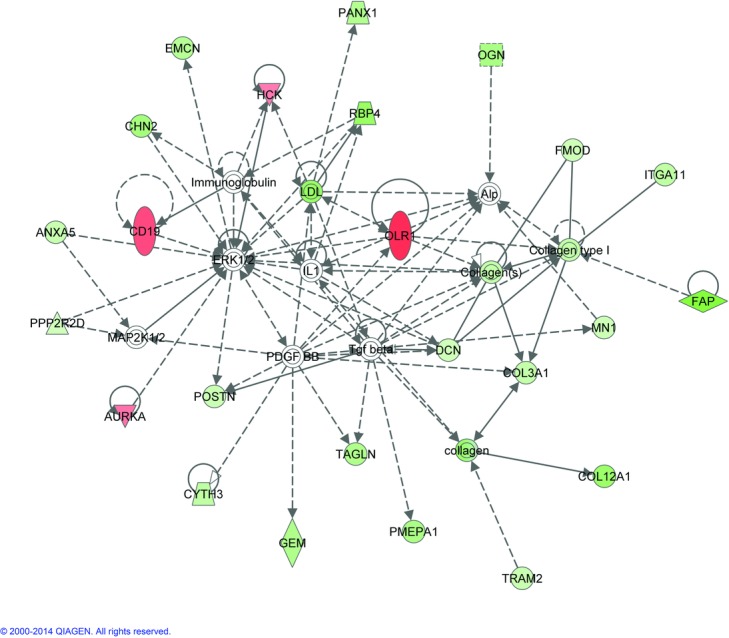
Pathway analysis. De novo pathway analysis using the false discovery rate (FDR) 0.15 gene list. Pathways were built with Ingenuity Pathway Analysis taking the genes with significant gene expressions changes from this experiment and identifying known relationships between the genes. Green indicates genes downregulated and red indicates upregulated genes from this study. The white genes did not have significant gene expression changes but link the genes in the network that are significantly changed. The intensity of the color indicates the degree of the expression change. Solid lines indicate direct interaction, while dashed lines indicate indirect interaction. Lines indicate binding between molecules, while arrows indicate molecule that can act on another which may or may not also bind.

FDR 0.10 and 0.15 gene lists and fold changes were evaluated through the Ingenuity Pathway Analysis (IPA) (Ingenuity® Systems, www.ingenuity.com) specifically de novo network analyses and predictions of upstream activators and inhibiters were most useful in analysis of the gene sets. The de novo network analysis used the gene lists derived from the microarray data from these experiments and found known relationships between the genes that created the pathways (Figs 1 and 2). Genes not on the list may be inserted into the pathways to make connections between the genes. Venny was used to create Venn diagrams to identify overlap of genes between studies.^7^^-^9^,^^12^ Chi-square analysis with Yates correction was used to determine the significance of overlapping genes (GraphPad).

## RESULTS

### Correlation between technical replicates

To test the accuracy and precision of the expression data generated from the Illumina BeadArray, we included technical replicates for 5 patent and 5 fused RNAs. RNA from a single isolation for each sample was divided into separate aliquots and processed through the complete Illumina assay, hybridized to a BeadArray, and scanned. Correlations between duplicate sample arrays were all greater than 0.95 (see Supplementary Table 1S). This suggests a high degree of precision providing an additional validation of our results.

### Differentially expressed genes

We conducted global gene expression analysis, using RNA from 7 pairs of fused and patent infant cranial suture samples in an effort to identify dysregulated genes. When comparing mRNA transcript expression in prematurely fused human infant cranial sutures to patent sutures, 68 genes were significantly differentially expressed with FDR 0.10 and fold changes ≤ −2.0 and ≥ 2.0; 5 were overexpressed while 63 were underexpressed. With relaxed FDR (0.15), 136 genes were significantly differentially expressed and had fold changes −2.0 or less and 2.0 or more; 21 had increased expression, while 114 showed decreased expression (Table 3).

The top-10 downregulated genes significant at FDR 0.10 and measured by fold change are shown in Table 4. With FDR relaxed to 0.15, *SFRP2*, *GPNMB*, *SCN2B*, *LRRC17*, *EDIL3*, and *FAP* are still included, but *DKK2*, *RORA*, *RGS22*, and *FGFBP2* are also in the top 10 significantly underexpressed with fold changes −2.0 or less. The gene lists are not identical since some of the genes added in the more relaxed criteria have greater fold changes than the genes they are displaced in the FDR 0.10 list. Identified genes with increased expression in synostosed cranial suture include *DOCK2*, *CDCA5*, *CDCA7*, *MOSC1*, and *CEACAM1* (Table 5). The top 10 significantly overexpressed genes at FDR 0.15 and having fold changes 2.0 or more include *EPB42*, *CCDC81*, *KEL*, *OLR1*, *CEACAM1*, *VPREB1*, *RAB3IP*, *HBQ1*, *CEACAM1* (alternative transcript), and *TRIM10*, a different list than FDR 0.10 except for *CEACAM1*.

To confirm gene expression changes, quantitative real-time PCR (qRT-PCR) was performed with 4 downregulated genes from our list, *SFRP2*, *GPNMB*, *MN1*, and *DKK2* using samples from patients 5 and 6. The amount of RNA from the remaining samples was not sufficient for validation studies and we did not validate these in independent samples due to the difficulty in obtaining additional tissue. *SFRP2*, *GPNMB*, and *DKK2* were all downregulated comparing patent to fused sutures in both samples (Supplemental Figure 1S and Supplemental Table 2S). *DKK2* was chosen to validate as it was identified with the more relaxed criteria; thus, FDR 0.15 genes may still be biologically relevant in craniosynostosis. *MN1* was identified in the FDR 0.01 data set but was only 2-fold downregulated according to the microarray. It was downregulated in patient 5 but slightly upregulated in patient 6. Additional samples would be necessary to determine whether *MN1* is generally downregulated as sutures fuse.

### Correlation of expression between patients

**Figure 3:**
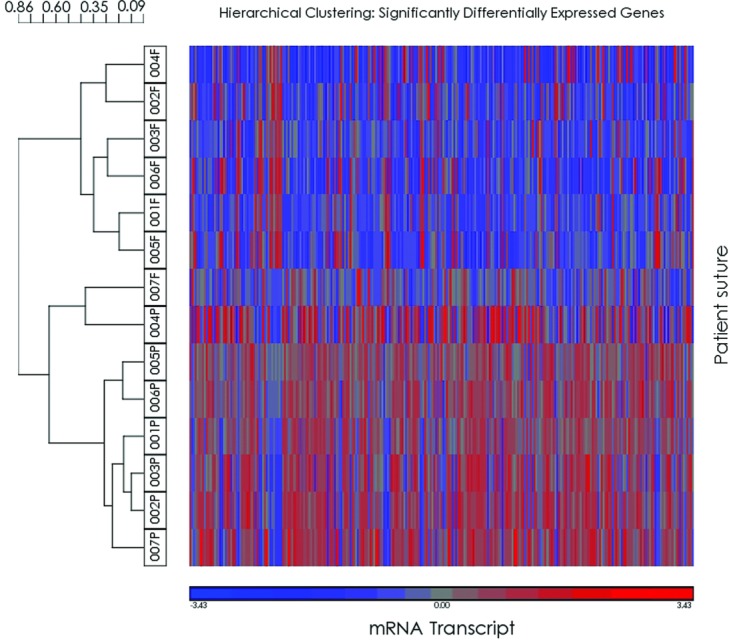
Heat map and hierarchical clustering. Clustering of individual suture samples using significantly differentially expressed genes. Individual suture samples are listed on the left with patient numbers (Table 1) and P referring to patent sutures and F to fused sutures.

Even though our samples were derived from only NSC individuals, we used different patent and fused suture type combinations in this study as well as samples from both male and female individuals. To determine whether this combination of different suture type tissue is an issue, we used hierarchical clustering with our significantly differentially expressed genes across samples (Fig 3). We found that in all but 1 case, the expression of the fused sutures cluster together as well as the patent suture irrespective of suture type or gender. In general, neither suture type nor sex clustered together within a group (patent vs fused). The 1 exception was individual 7, whose fused suture clustered with the patent suture group most closely with patent suture from patient 4, the only sagittal patent suture studied.

**Figure 4:**
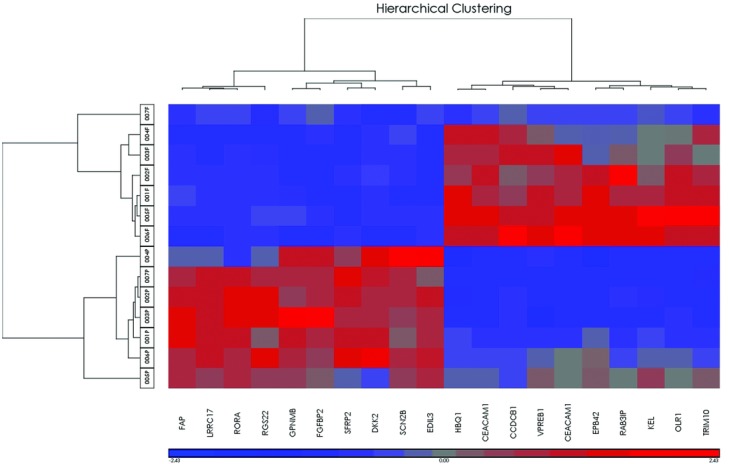
Heat map and hierarchical clustering. Clustering of individual suture samples and top 10 genes significantly upregulated and downregulated genes. Individual suture samples are listed on the left with patient numbers (Table 1) and P referring to patent sutures and F to fused sutures.

To further identify the molecular signature of NSC and genes most important in maintaining patency across suture type and sex, we refined our gene list to the top-10 significantly upregulated and downregulated transcript probe sets for the entire sample. Hierarchical clustering as conducted using log 2 transformed normalized signal for only these probe sets to create a heat map and dendrogram (Fig 4). Both samples and genes were clustered. Measure of dissimilarity was Pearson's dissimilarity with complete linkage. Using only the top-10 up- and downregulated genes 2 major branches separated fused and patent samples. Within suture status, sex and suture type did not influence clustering. Suture expression represented by log 2 transformed signal converted to *z* score with mean of 0 and SD of 1 clearly delineates patent from fused. Thus, our data show that we can identify genes that are generally changed between patent and fused sutures, irrespective of sex or suture type.

### Differentially expressed genes in sagittal sutures only

Results of paired *t* test considering sagittal suture synostosis only resulted in 2 significantly differentially expressed genes when implementing FDR 0.10 with *P* ≥ .05 and fold change stringency −2.0 or less and 2.0 or more (Table 2S). With relaxed FDR to 0.15, 119 genes were significant and had fold changes −2.0 or less and 2.0 or more; 114 were downregulated in prematurely fused sagittal suture while 6 demonstrated increased expression (Supplemental Table 3S). *SFRP2* and *SPHKAP* were downregulated and significant at FDR 0.10. Interestingly, *SPHKAP* is not identified in the original analysis, perhaps indicating a gene important in sagittal suture fusion. Considering genes significant with FDR 0.15, there were only 6 upregulated genes, which include *APOBEC3F*, *AMMECR1*, *IKZF3*, *MYO3A*, *OR10H5*, and *IRE4*. The top-10 downregulated genes were *B4GALT4*, *C10ORF26*, *CCD074A*, *EPHB2*, *GPC3*, *LAMA2, RORA*, and *SFRP2*.

Comparing the sagittal only gene set with the entire set reveals that *SFRP2* is significantly downregulated in prematurely fused suture across the entire set at FDR 0.10 (fold changes ≤ −2.0). This suggests that *SFRP2* has a significant role as a universal mechanism in maintaining suture patency. Considering genes differentially expressed with the relaxed FDR, 35 genes are found in common. These genes likely represent a set of genes important in suture patency across all sutures.

### Gene functions

To determine whether the gene expression data have biological meaning, the significant genes in both FDR 0.10 and 0.15 sets were analyzed through the Spring 2013 release of Ingenuity Pathway Analysis including Upstream Analysis, which identifies upstream regulators that can explain the observed gene expression changes (Ingenuity® Systems, www.ingenuity.com). To test if it is valid to include genes with less stringent criteria (FDR 0.15), we see that the significant canonical pathways overlap in the 2 groups of genes (Table 6). Both gene lists (FDR 0.10 and FDR 0.15) identified the same canonical pathway (osteoblasts, osteoclasts, and chondrocytes in rheumatoid arthritis). In fact, it is the only significant canonical pathway at FDR 0.15, which justifies including this list of genes. The identification of these specific pathways would be expected in genes important in suture biology.

The top de novo networks generated with the 2 different data sets connect to interesting cellular signaling genes (Figs 1 and 2). Genes in both pathways include *ERK1/2* and *PDGFBB*. The pathway created with FDR 0.10 gene list also include *p38 MAPK, Akt*, and *IRS1*, while the top network with FDR 0.15 genes also includes *TGFβ*, all genes important in cellular morphology, cell assembly and organization, connective tissue, and known to be important in suture biology.

Finally, we looked at putative upstream regulators of these differentially expressed genes (Table 7). Interestingly, the most significant molecule and the ability to predict how the molecule might be regulated (*z* scores) in both gene sets is the inhibition of *WNT3A*. The only other change found in both lists is the inhibition of *TGFβ1*. It seems that in NCS Wnt and TGFβ signaling might be involved in suture patency and FGFs may not be the activator upstream of suture closure in NS cases.

### Genes across studies

**Figure 5:**
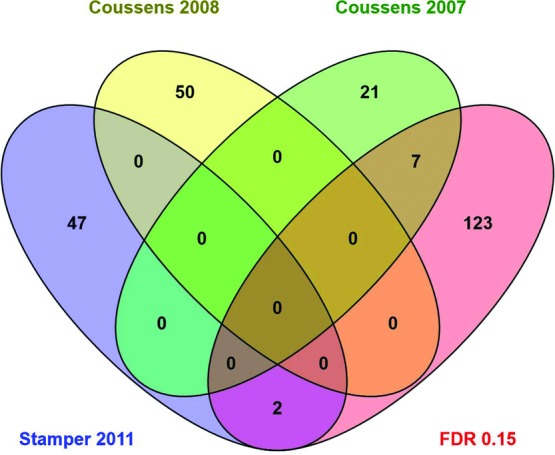
Venn diagram. Overlap of the number of genes identified as significantly differentially expressed (FDR 0.15) in this study and other studies.^7^^-^9

To determine whether our gene list corroborates previous studies, we compared the gene list from this study with genes identified in 3 previous human studies (Fig 5). First, we compared our gene list with study results of differentially expressed genes between fused sutures and suture tissue that were dedifferentiated in culture.^7^ There was no overlap with our most relaxed gene list (FDR 0.15) with the top-50 genes from that study. A large number of differentially expressed genes were identified in that study (∼2000) so the genes identified from this study could have overlapped with the genes in the larger list. The second list was of single sutures with tissue also grown in tissue culture and compared to a set of unrelated controls.^9^ Again, only the top genes from that study were analyzed (47 genes) and 2 genes overlap with our FDR 0.15 gene list, *GEM, MAB21L2* (*P* = .007) (Fig 5). Interestingly, comparing the gene list from this study with a much smaller list of significantly differentially expressed genes (28 genes) using primary suture tissue, 6 genes in common were identified with the FDR 0.10 list and include *FBLN1, FMOD, MFAP4, MN1, RBP4, SPON1* (*P* < .0001) (data not shown).^8^ One additional gene can be included using a more relaxed FDR 0.15 (*PDZRN3*) (*P* < .0001) (Fig 5). Our study identifies 25% of their significantly changed genes, which supports the importance of these genes in suture patency and that these downstream changes seem to be irrespective of the primary cause of craniosynostosis in these cases (Fig 5). There was no overlap between gene lists between any of the previously published studies.

## DISCUSSION

In this study, microarray analysis was used to study differential gene expression between patent and fused sutures in individuals with a nonsyndromic form of craniosynostosis using only primary suture tissue. These individuals did not carry any of the known syndromic mutations. The majority of the samples were fused sagittal sutures paired with patent lambdoid sutures. A coronal/sagittal and metopic/coronal pair was also included. Even though we were able to use fused and patent samples from the same individual we were not able to obtain fused and patent samples from the same sutures in the same individual. This would only be possible in cases of unilateral coronal or lambdoid craniosynostosis, and for the single unilateral coronal case included in this study, the patent sample obtained was derived from the sagittal suture. These unilateral cases are also quite rare and it would be difficult to obtain enough cases for this study. We could have paired patent and fused tissue from the same suture type obtained from different individuals but gene expression changes may then have reflected genetic difference between individuals. This is a major limitation of the study as we are making the assumption that gene expression differences between patent and closed sutures of different suture types behave similarly. To address this issue, we conducted hierarchical clustering of individual samples, using normalized and standardized signal intensity data to retrospectively test appropriateness of our experimental design. Clustering of samples by patient pair, fused or patent suture status, or suture type would suggest that these factors have a greater effect than suture status and therefore may mask genes dysregulated in synostosed suture. Hierarchical clustering showed that patent and fused sutures clustered with each other, irrespective of suture type or pair except in 1 case of a fused sagittal suture (patient 7) clustering with the patent sutures. Therefore, gene differences from our study design would identify gene expression changes affecting suture patency and closure across suture types.

The use of completely fused sutures instead of sutures in the process of fusing in this study reveals the final gene expression changes that result in premature fusion. The advantage is that across different samples they are at the same developmental stage whereas fusing sutures could be at different stages of fusion. In addition, predictions can be made of what is activated upstream of gene changes identified at a terminal developmental time point. It unfortunately does not capture what is happening on the osteogenic front as the sutures are closing.

The 4 genes that were chosen for validation included 2 genes that had large gene expression changes and significance at an FDR level of 0.01, *SRFP2* (−10.96) and *GPNMB* (−8.59) (Tables 1 and 2). *MN1* was chosen as it was also significant at an FDR level of 0.01 but had a much smaller fold change of −2.27 (Table 1). Finally, *DKK2* was validated as it was not as significant (FDR 0.15 gene list) but also had a large gene expression change, −9.74, by microarray (Tables 1). *SRFP2*, *GPNMB*, and *DKK2* were shown to be downregulated in fused versus patent sutures similar to the microarray. This shows that genes included when the FDR rate was raised from 0.10 to 0.15 may have real changes of biological importance. The *MN1* gene was downregulated in patient 5 but not in patient 6. Thus, this gene expression change could not be validated and more studies should be initiated because 1 other gene expression study found *MN1* to be differentially regulated.^8^ The fold changes were much greater in the validation studies of *SFRP2* and *DKK2* than by microarray analysis. This is not unexpected as differences in fold change magnitude are often observed when comparing microarray and qRT-PCR results, with qRT-PCR being larger.^13^^-^18 However, despite this, fold changes of the same direction observed in qRT-PCR relative to microarray are accepted as concordant and confirmatory.^13^^-^18 Several factors contribute to the differences, including greater sensitivity and dynamic range for the qRT-PCR platform that can accurately perform with up to 1000-fold less RNA than microarray.^16^^-^18 The microarray platform is less sensitive and has a narrower dynamic range.

### Wnt signaling

Wnt signaling plays an important role in bone development and it is well known that an increase in Wnt signaling increases bone mineralization (reviewed in Baron and Kneissel^19^). In contrast to mature bone mineralization, when specifically investigating Wnt signaling in suture closure in mice, continual Wnt signaling is necessary for suture patency in sagittal sutures through the downregulation of *TWIST*.^20^ *Wnt3a* inhibition is predicted by Ingenuity Pathway Analysis to be upstream of gene expression changes from this study and seems to reinforce the findings of Behr et al.^20^ Specifically, *Wnt3a* has been shown to inhibit chondrogenesis through the reorganization of the cytoskeleton and prevent osteogenic differentiation by promoting proliferation.^21^^-^23

*SFRP2*, a secreted frizzled-related protein, was identified as an important gene downregulated in fused sutures as it is significantly decreased in fused tissue in all data sets (0.10 FDR, 0.15 FDR, and sagittal only 0.10 FDR and 0.15 FDR). Another *Wnt/β-catenin* pathway gene is also significantly downregulated, *DKK2* (top 10 in FDR 0.15). *DKK2* is involved in terminal osteoblast differentiation. Both *SFRP2* and *DKK2* were validated with qRT-PCR and have been identified as negative regulators of canonical and noncanonical *Wnt/β catenin* signaling pathway. One would expect that increased canonical *Wnt* signaling would result if negative regulators of *Wnt* signaling were downregulated. It is likely to be more complicated. *DKK2* can be an agonist or antagonist to Wnt signaling. *DKK2* can activate Wnt signaling through the LRP6 receptor.^24^ In the vertebrate optic cup, *SFRP1* and 2 was shown to positively modulate canonical Wnt signaling by helping diffuse Wnt through the tissue and bringing it into close proximity of its receptor.^25^ Thus, downregulation of *SFRP2* and *DKK2* does not necessarily indicate an increase in Wnt signaling since *SFRP2* and *DKK2* can either antagonize or positively modulate Wnt signaling.

### Additional differentially expressed genes and their involvement in craniosynostosis

Other top-10 downregulated genes include *GPNMB*, *LRRC17*, *EDIL3*, *FAP*, *LRIG3*, and *GDF10* (FDR 0.15). These genes are known to be involved in osteogenesis, cell adhesion, and invasion or angiogenesis. For example, *LRRC1* acts as a negative regulator of RANKL-induced (receptor activator of NF-κB ligand) osteoclast differentiation.^26^ Blood vessel formation is known to be an important aspect of bone formation. The 4 additional downregulated genes in the top-10 list (*SCN2B*, *FOXP2*, *PCDH19*, and *RBP4*) all are found to be expressed in the brain or have brain phenotypes. Interestingly, signals from the dura and brain development are also thought to affect suture biology.

The top upregulated genes in the FDR 0.10 and FDR 0.15 lists are not known to be important in bone formation or suture biology. They are involved in the endothelial cells, hematological tissues, and important in cancer. These are candidate genes that may be found to be important in suture biology.

### Sagittal suture synostosis

*SPHKAP* was identified as a significantly downregulated gene in the analysis of only fused sagittal sutures that may indicate the importance of this gene in preserving sagittal suture patency. This gene codes for a type I A-kinase anchoring protein and has been shown to interact with and modulate sphingosine kinase activity (*SPK1*).^27^ *SPK1* mediates *TGFβ* signaling of extracellular matrix deposition and maintenance.^28^ The extracellular matrix contains components that can signal through receptors that control proliferation, migration, differentiation, and apoptosis. In a recent genome-wide association study (GWAS) using trios with children with sagittal craniosynostosis, 2 regions of the genome that were associated with this phenotype were *BMP2* and *BBS9*.^29^ *BMP2* is a *TGFβ* family member. Although no known connection between *SPHKAP* and *BMP2* has been observed, it will be interesting to determine whether *SPHKAP* has any effect on the signaling of *BMP2* specifically in sagittal sutures.

### Genes identified across studies

There have been a variety of studies that have identified differentially expressed genes between patent and fused sutures. Most studies included tissue from individuals with known syndromic mutations in their study population; others cultured the osteoblasts prior to gene expression analysis. In addition, different experimental platforms and different criteria were used to generate gene lists. This makes it difficult to compare gene lists across studies. We looked at gene lists from 3 additional human studies^7^^-^9 and there was no overlap using their short gene lists between the previous studies but we had significant overlap with 7 genes with the study that also used primary suture tissue as their starting material^8^ and 2 genes with a study with cultured osteoblast. These 9 genes, *GEM*, *MAB21L2*, *FBLN1*, *FMOD*, *MFAP4*, *MN1*, *RBP4*, *SPON1*, and *PDZRN3*, are likely very important across suture type and irrespective of the cause of craniosynostosis.

*FBNL1*, *FMOD*, *MFAP4*, and *SPONL* are all proteins that are found in the extracellular matrix. The extracellular matrix not only is an exceedingly important component of bone formation as a structural component but also plays a role in cell signaling. For example, *FMOD* is a small proteoglycan that can bind collagen and inhibit fibrillogenesis as well as has the ability to sequester grow factors such as *TGFβ* in the extracellular matrix.^30^^,^^31^ Two of the genes, *MN1* and *PDZRN3*, are known to be important in osteoblast induction or function. Gene ontology revealed that *MN1* has a critical function in intramembranous ossification. *MN1* is the gene that was validated only in 1 out of 2 samples by qRT-PCR that would not preclude its importance in craniosynostosis in some cases. *MN1* is a transcription coregulator and studies show that *MN1* is necessary to maintain appropriate osteoblast proliferation, differentiation, and function, including support of osteoclastogenesis.^32^ *PDZRN3* is necessary for the negative feedback loop of *BMP2*-induced osteoblast differentiation. This feedback loop utilizes the canonical *Wnt* signaling pathway.^33^

### Signaling networks and activators predicted to be involved in craniosynostosis

To identify upstream activators or other genes related to our genes lists, IPA analysis was used to create *de novo* networks and to identify putative upstream activators (Figs 3 and 4 and Table 5). Signaling molecules such as *p38/MAPK, PI3K/Akt, ERK1/2, PDGF-BB* and *TGFβ* were included with genes from our gene list to create our top gene networks. Constitutively active *FGFRs* are known to cause craniosynostosis and FGFRs also signal through *p38/MAPK, PI3K/Akt* and *ERK1/2*. Our samples have no known *FGFR* mutations, but similar downstream pathways seem involved in this craniosynostosis cohort. We do not know if there are changes in any *FGF* molecules but other upstream signals can affect these molecules. Activation of *PI3K/Akt* and *ERK1/2* can be achieved through *TGFβ*. *PDGF-BB* can also signal through *p38/MAPK*, *PI3K/Akt*, and *ERK1/2*. *TGFβ1* and *3* and *Wnt3a* were identified as putative upstream molecules (Table 5). Both are known to be important in bone formation.

## CONCLUSIONS

We have identified a set of genes that are differentially expressed between patent and fused sutures in a NSC sample, using mixed sutures and primary tissue. We identified *SFRP2* as differentially expressed in all analyses and likely to be important in suture patency. Analysis with sagittal only sutures identified a set of 35 overlapping genes that may be involved in suture patency over all suture types. *SPHKAP* was identified in only the sagittal subset and may be specific for that particular suture type. In addition, we found overlap of 7 genes in our study with that of a previous study highlighting the importance of those genes regardless of the etiology of the craniosynostosis. In addition, the same signaling pathways known to be important in suture patency and bone formation seem to be activated in suture fusion again, irrespective of etiology. The identification of genes associated with suture biology will also aid in the identification of genes involved in this disorder. With the advent of next-generation sequencing, genes identified to have suture involvement become candidates in these studies if variants are identified in these genes.

## AVAILABILITY OF SUPPORTING DATA

The data set supporting the results of this article are available in the Gene Expression Omnibus repository, accession number and link to data set GSE50796.

## Figures and Tables

**Table 1:** Patient information

Patient	Patent suture sample	Fused suture sample	Sex	Age at surgery, mos
1	Lambdoid	Sagittal	M	4
2	Lambdoid	Sagittal	M	4
3	Lambdoid	Sagittal	F	4
4	Sagittal	Coronal	F	10
5	Coronal	Metopic	M	10
6	Lambdoid	Sagittal	F	4
7	Lambdoid	Sagittal	F	4

**Table 2:** Quantitative real-time PCR primers

Gene	Forward primer	Reverse primer	Amplicon size, bp
*DKK2*	GTACCAAGGACTGGCATTCG	TGGCAATACCTCCCAACTTC	102
*GPNMB*	GAAGGTCTTTCATTGCCCAG	CGTGAGAATTCAGCATGGAA	118
*MN1*	GCAGTGGACAGACAGGCAC	CTGGGAGAAGGCCAAACC	115
*SFRP2*	CAACGACATAATGGAAACGC	TGGTCTTGCTCTTGGTCTCC	116
*18S*	TTCGGAACTGAGGCCATGAT	CGAACCTCCGACTTTCGTTCT	151

**Table 3:** Significantly differentially expressed genes, *P* < .05 FDR 0.10 and 0.15*

Column ID	Adjusted *P* value	Mean (fused)	Standard deviation (fused)	Mean (patent)	Standard deviation (patent)	Fold change (fused/patent)
*SFRP2*	.08	298.257	206.94	3268.56	906.61	−10.96
*DKK2*	.13	137.671	85.06	1340.51	449.33	−9.74
*RORA*	.15	313.914	240.58	3016.7	1265.91	−9.61
*RGS22*	.13	58.2143	35.37	513.571	174.27	−8.82
*GPNMB*	.09	215.114	215.59	1848.66	378.81	−8.59
*SCN2B*	.10	145.514	105.03	1014.14	380.25	−6.97
*LRRC17*	.09	737.357	634.80	4865.97	1415.69	−6.60
*EDIL3*	.10	150.357	111.78	966.329	196.99	−6.43
*FAP*	.08	3382.17	2683.98	21488.5	6025.73	−6.35
*FGFBP2*	.14	896.157	817.10	5611.4	1707.62	−6.26
*LRIG3*	.10	572.643	716.58	3538.51	865.86	−6.18
*FOXP2*	.09	159.886	94.64	888.071	239.25	−5.55
*PCDH19*	.05	372.114	164.83	2023.09	248.63	−5.44
*BCL11B*	.15	785.514	455.85	3983.14	1176.17	−5.07
*RBP4*	.08	781.814	440.85	3918.19	696.23	−5.01
*WNT16*	.09	321.843	257.28	1604.67	541.81	−4.99
*SEPT10*	.09	585.443	410.77	2881.37	727.87	−4.92
*COL12A1*	.09	1613.34	1750.94	7696.4	1717.96	−4.77
*IGSF10*	.06	482.957	175.22	2275.23	312.04	−4.71
*GOLM1*	.09	459.086	261.64	2132.1	323.63	−4.64
*QKI*	.05	645.329	555.78	2977.96	363.91	−4.61
*CA11*	.11	339.986	258.82	1532.96	445.88	−4.51
*PDZRN3*	.14	561.857	465.69	2519.93	574.57	−4.49
*KAL1*	.12	238.686	204.96	1065.07	338.86	−4.46
*VWA5A*	.09	202.371	161.53	894.6	202.35	−4.42
*MEX3B*	.09	503.857	346.05	2214.27	200.22	−4.39
*GPNMB (alt.transcript)*	.05	536.129	302.20	2340.86	238.59	−4.37
*PID1*	.09	543.1	354.77	2334.17	482.23	−4.30
*CHN2*	.05	702.9	272.08	2907.7	222.49	−4.14
*FBXO32*	.11	553.886	370.76	2253.01	545.72	−4.07
*C2ORF32*	.08	852.086	598.05	3399.77	501.03	−3.99
*GDF10*	.11	447.786	336.17	1781.09	362.30	−3.98
*MATN2*	.10	292.243	172.37	1148.34	195.31	−3.93
*OGN*	.11	555.157	405.95	2155.31	291.80	−3.88
*PMEPA1*	.13	569.857	306.84	2208.07	709.51	−3.87
*MT1M*	.09	659.443	319.17	2523.56	731.93	−3.83
*PGCP*	.15	436.029	404.11	1638.77	304.53	−3.76
*GEM*	.10	886.757	495.78	3239.1	631.27	−3.65
*SNX7*	.09	1324.1	940.19	4759.29	898.77	−3.59
*TAGLN*	.09	227.643	109.34	817.7	150.32	−3.59
*TSC22D1*	.05	560.429	109.34	2007.8	150.32	−3.58
*SIX2*	.09	284.014	479.62	1012.71	407.01	−3.57
*MFAP4*	.08	3961.47	207.40	13877.2	288.98	−3.50
*PANX1*	.14	193.057	2020.27	665.5	1402.53	−3.45
*C3ORF14*	.10	1456.93	192.52	4957.09	221.32	−3.40
*SPOCK1*	.13	1809.79	1307.51	6121.91	1299.05	−3.38
*EMCN*	.13	417.757	370.86	1412.61	134.58	−3.38
*TMEM39A*	.05	828.543	332.69	2794.51	297.01	−3.37
*TMTC1*	.14	85.2	57.72	286.229	88.04	−3.36
*CRTAC1*	.10	660.586	312.53	2208.54	698.58	−3.34
*FBLN1*	.08	4812.31	2403.24	16054.9	1754.02	−3.34
*ZBTB38*	.10	133.7	83.98	442.829	100.74	−3.31
*HECTD2*	.14	879.114	440.69	2876.6	543.53	−3.27
*NNAT*	.05	1098.91	405.57	3512.13	724.37	−3.20
*DCLK1*	.14	717.771	412.96	2285.34	385.73	−3.18
*FAM114A1*	.10	394.343	109.23	1217.36	338.54	−3.09
*DCTD*	.09	697.043	503.22	2140.27	102.41	−3.07
*MAP4K3*	.13	850.014	778.89	2608.29	776.60	−3.07
*ATRN*	.13	482.6	398.95	1445.44	167.19	−3.00
*C2ORF40*	.11	1676.94	728.41	5022.61	1394.66	−3.00
*MAB21L2*	.13	2312.51	1363.46	6837.99	1304.40	−2.96
*CYTH3*	.15	537.129	1327.53	1552.8	1406.00	−2.89
*ITGA11*	.10	1875.94	101.70	5379.17	101.54	−2.87
*FAT4*	.07	297.957	104.06	846.343	119.88	−2.84
*B3GALNT1*	.10	289.729	607.14	818.714	467.53	−2.83
*BMP8B*	.08	826.043	165.53	2271.23	150.36	−2.75
*RNF180*	.08	222.586	846.29	608.386	603.74	−2.73
*PTPRK*	.10	2043.43	2878.61	5488.21	1304.93	−2.69
*COL3A1*	.08	5017.29	102.17	13455.4	75.54	−2.68
*USP46*	.09	237.786	612.75	634.771	334.41	−2.67
*S100A16*	.10	1112.33	279.17	2915.74	204.32	−2.62
*USP21*	.14	325.629	233.17	847.657	334.27	−2.60
*RASAL2*	.08	467.8	1668.92	1200.71	2475.17	−2.57
*TPM1*	.09	3458.41	3270.79	8833.56	3186.75	−2.55
*ANXA5*	.10	5406.47	249.97	13798.4	276.27	−2.55
*QKI*	.14	420.9	2699.23	1062.8	987.62	−2.53
*PDGFRL*	.08	7487.53	210.60	18865.4	143.82	−2.52
*C21ORF34*	.13	392.243	495.78	982.4	631.27	−2.50
*AQP1*	.15	476.971	319.81	1166.54	409.73	−2.45
*PAM*	.15	6074.54	2719.12	14817.1	1540.54	−2.44
*LRRC66*	.15	118.243	40.54	288.086	56.64	−2.44
*MYO1E*	.14	261.657	119.82	634.686	167.12	−2.43
*BET1L*	.13	534.7	253.64	1291.19	244.62	−2.41
*TRAM2*	.14	1162.84	777.40	2806.31	447.56	−2.41
*TPM1*	.09	1437.84	905.49	3449.71	529.71	−2.40
*TENC1*	.13	473.757	277.33	1129.69	252.88	−2.38
*POSTN*	.12	2669.66	1405.11	6359.14	805.24	−2.38
*PDPN*	.11	479.543	506.99	1116.77	385.41	−2.33
*DLX5*	.14	3177.21	1910.37	7368.31	1661.00	−2.32
*C7ORF53*	.14	214.457	149.16	496.643	93.43	−2.32
*HNRPDL*	.12	2890.2	1373.31	6642.73	1452.84	−2.30
*TWF1*	.10	777	262.76	1780.44	347.67	−2.29
*MN1*	.10	2680.2	920.26	6084.87	458.25	−2.27
*FMOD*	.09	2203.19	984.06	4989.59	695.63	−2.26
*GRAMD3*	.14	1750.54	954.59	3936.4	296.69	−2.25
*DYNC1I1*	.12	2979.24	1111.06	6570.27	752.26	−2.21
*GREM2*	.15	765.757	526.55	1688.54	483.93	−2.21
*LRRC1*	.13	224.086	131.06	491.143	73.63	−2.19
*CPXM2*	.08	2268.56	842.46	4964.26	758.28	−2.19
*DCN*	.14	5608.17	2717.98	12108.9	2527.47	−2.16
*TSHZ1*	.09	1208.16	419.49	2581.64	262.97	−2.14
*COL11A1*	.07	2717.17	1433.14	5790.13	974.38	−2.13
*RNASE4*	.08	3780.09	672.89	8037	848.05	−2.13
*TCEAL2*	.14	4380.21	1902.65	9248.66	1870.95	−2.11
*CASK*	.13	2360.27	741.13	4951.31	1000.32	−2.10
*PPP2R2D*	.14	222	122.74	465.629	62.11	−2.10
*CGNL1*	.14	3326	1399.42	6829.57	1148.31	−2.05
*RYK*	.07	1740.41	478.61	3527.83	628.86	−2.03
*SPON1*	.05	3446.23	1042.93	6965.39	533.25	−2.02
*CLIC4*	.13	843.971	331.25	1704.27	125.02	−2.02
*LIMA1*	.15	2363.43	1135.43	4768.59	908.80	−2.02
*ANTXR1*	.11	1860.01	479.20	3750.5	419.39	−2.02
*TM9SF1*	.10	944.943	441.28	1903.8	246.34	−2.01
*IRS1*	.09	1788.3	671.67	3582	368.95	−2.00
*HCK*	.13	9185.66	3558.93	4579.29	2238.88	2.01
*DOCK2*	.09	5023.77	1778.63	2411.3	1315.08	2.08
*AURKA*	.12	5988.07	2174.27	2772.26	1253.44	2.16
*CDCA5*	.10	6840.26	1729.70	3164.04	757.04	2.16
*ITGAM*	.11	2333.57	1177.15	1038.93	786.43	2.25
*AMMECR1*	.11	809.257	287.97	328.429	155.42	2.46
*CENPF*	.15	8258.67	2486.46	3229.84	868.51	2.56
*CDCA7*	.09	5903.33	1970.28	2275.77	1302.15	2.59
*MARC1*	.09	3007.17	1047.21	1132.36	649.43	2.66
*FCRL2*	.12	2463.39	719.52	877.457	590.05	2.81
*CD19*	.14	9667.1	3120.49	3414.04	2411.22	2.83
*LILRA3*	.15	5095.6	2098.19	1790.16	1012.29	2.85
*EPB42*	.13	6131.57	2776.63	2113.6	1873.53	2.90
*CCDC81*	.13	1387.86	478.69	412.343	116.40	3.37
*KEL*	.11	5061.03	2700.28	1483.19	1533.03	3.41
*OLR1*	.11	4177.71	1575.20	1214.81	934.13	3.44
*CEACAM1 (alt.transcript)*	.13	4430.7	1761.43	1267.56	1017.67	3.50
*VPREB1*	.11	15753.4	4840.54	4498.87	4238.12	3.50
*RAB3IP*	.15	4386.47	1879.28	1227.57	861.14	3.57
*HBQ1*	.15	4088.11	1364.49	1046.96	744.91	3.90
*CEACAM1*	.10	3893.66	1761.43	950.2	1017.67	4.10
*TRIM10*	.13	3067.09	1348.32	744.571	791.54	4.12

*Adjusted *P* value—Benjamini and Hochberg correction for multiple testing.

**Table 4:** Top 10 downregulated genes with FDR ≤ 0.10*

Gene	Adjusted *P* value	Mean (fused)	Standard deviation	Mean (patent)	Standard deviation	Fold change (fused/patent)
*SFRP2*	.08	298.257	206.94	3268.56	906.61	−10.96
*GPNMB*	.09	215.114	215.59	1848.66	378.81	−8.59
*SCN2B*	.10	145.514	105.03	1014.14	380.25	−6.97
*LRRC17*	.09	737.357	634.80	4865.97	1415.69	−6.60
*EDIL3*	.10	150.357	111.78	966.329	196.99	−6.43
*FAP*	.08	3382.17	2683.98	21488.5	6025.73	−6.35
*LRIG3*	.10	572.643	572.643	3538.51	865.86	−6.18
*FOXP2*	.09	159.886	94.64	888.071	239.25	−5.55
*PCDH19*	.05	372.114	164.83	2023.09	248.63	−5.44
*RBP4*	.08	781.814	440.85	3918.19	696.23	−5.01

*Adjusted *P* value—Benjamini and Hochberg correction for multiple testing. FDR indicates false discovery rate.

**Table 5:** Top upregulated genes with FDR ≤ 0.10*

Gene	Adjusted *P* value	Mean (fused)	Standard deviation	Mean (patent)	Standard deviation	Fold change (fused/patent)
*DOCK2*	.09	5023.77	1778.63	2411.3	1315.08	2.08
*CDCA5*	.10	6840.26	1729.70	3164.04	757.04	2.16
*CDCA7*	.09	5903.33	1970.28	2275.77	1302.15	2.59
*MARC1*	.09	3007.17	1047.21	1132.36	649.43	2.66
*CEACAM1*	.10	3893.66	1761.43	950.2	1017.67	4.10

*Adjusted *P* value—Benjamini and Hochberg correction for multiple testing. FDR indicates false discovery rate.

**Table 6:** Significant canonical pathways*

FDR 0.10		FDR 0.15	
Canonical pathways	*P*	Canonical pathways	*P*
Basal cell carcinoma signaling	.03	Role of osteoblasts, osteoclasts and chondrocytes in rheumatoid arthritis	.02
Role of osteoblasts, osteoclasts and chondrocytes in rheumatoid arthritis	.05		

*FDR indicates false discovery rate.

**Table 7:** Putative upstream regulators*

Upstream regulator	Predicted activation state	Activation *z*-score	*P* value of overlap
**FDR 0.10**			
*WNT3A*	Inhibited	−2.2	4.43E-04
*PRL*	Inhibited	−2.1	5.71E-04
*TGFB1*	Inhibited	−2.2	5.71E-03
**FDR 0.15**			
*WNT3A*	Inhibited	−3.0	4.30E-06
*TGFB1*	Inhibited	−2.3	8.47E-06
*HRAS*	Activated	2.2	9.05E-04
*SMAD7*	Activated	2.2	9.50E-04
*phosphate*	Activated	2.0	2.57E-03
*AHR*	Activated	2.4	3.82E-03
*ARNT2*	Inhibited	−2.0	4.44E-02
*SIM1*	Inhibited	−2.0	4.84E-02

*FDR indicates false discovery rate. Activation z scores indicate how well the downstream genes are changed in the predicted direction of the activation state.
